# Antitumor Effects and Related Mechanisms of Ethyl Acetate Extracts of *Polygonum perfoliatum* L.

**DOI:** 10.3389/fonc.2019.00578

**Published:** 2019-07-04

**Authors:** Qinglin Li, Xiaoxuan Fu, Xinyang Ge, Feng Tao, Ping Huang, Minghua Ge, Hongchuan Jin

**Affiliations:** ^1^Sir Run Run Shaw Hospital, Medical School of Zhejiang University, Hangzhou, China; ^2^Zhejiang Cancer Hospital, Hangzhou, China; ^3^Heartland Christian School, Columbiana, OH, United States; ^4^College of Pharmacy, Hangzhou Medical College, Hangzhou, China

**Keywords:** antitumor, effects, mechanisms, *Polygonum perfoliatum* L., PEC

## Abstract

*Polygonum perfoliatum* L. belongs to the genus *Polygonaceae* and has a long history to be used as a Chinese medicinal herb to reduce swelling, control body temperature, and promote detoxification. However, its anticancer activity and mechanisms of action have not been evaluated yet. In the present study, we used several cell lines and xenograft models from different cancers to demonstrate the broad-spectrum anticancer activity of *P. perfoliatum* L as well as its underlying mechanisms of action *in vitro* and *in vivo*. The ethyl acetate extract of *P. perfoliatum* L showed good anticancer activity and was further fractioned to obtain five active components, including PEA to PEE. Among these fractions, PEC showed the strongest cytotoxicities against various cancer cell lines. It was further observed that PEC inhibited cancer cell growth, arrested cells at G2 phase, and induced apoptosis *in vitro* and suppressed tumor growth and angiogenesis *in vivo* in a dose- and time-dependent manner. Furthermore, PEC decreased the expression of vascular endothelial growth factor (VEGF) and micro-vascular density (MVD) in tumor tissues *in vivo*. It also promoted the proliferation of T and B lymphocytes, increased the activities of natural killer (NK) cells and cytotoxic T lymphocytes (CTLs), enhanced the secretion of interleukin 2 (IL-2) by spleen cells, and raised the levels of IgG, IgG2a, and IgG2b antibodies in tumor-bearing mice *in vivo*, which were at least partially responsible for the anticancer activity of PEC. In summary, PEC has shown broad-spectrum anticancer activities without causing any host toxicity *in vitro* and *in vivo* and may be developed as a preventive and therapeutic agent against human cancer. Further studies are urgently needed to determine the anticancer compounds in PEC and their detailed molecular mechanisms.

## Introduction

*Polygonum perfoliatum* L. is a Chinese herb with high medicinal value and considered to have antipyretic and detoxification properties. It is used as a traditional Chinese medicine due to its ability to reduce swelling, control body temperature, and promote detoxification. Modern pharmacological studies on *P. perfoliatum* L. have confirmed that it exerts anti-inflammatory and antibacterial effects (1–3). Flavonoids and triterpene acids were identified as the active components that are responsible for the medicinal attributes of *P. perfoliatum* L (4). However, its anticancer activity and mechanisms of action have not been reported yet. Our research team obtained four kinds of extracts from *P. perfoliatum* L. using petroleum ether, ethyl acetate, ethanol, and water, respectively. The ethyl acetate extract showed the strongest activities among the four crude extracts and was further chromatographed and evaluated, resulting in the most active component PEC. In the present study, we further investigated the *in vitro* and *in vivo* anticancer activities effects of PEC and the underlying mechanisms.

## Materials and Methods

### Cell Line

Human cervical cancer cell line (Hela), human gastric cancer cell line (SGC-7901), human prostate cancer cell line (PC-3), human lung cancer cell line (A549), human glioma cell line (BT-325), and human pancreatic cancer cell line (PANC-1) were all provided by the Institute of Pharmaceutical Research of Zhejiang Academy of Medical Sciences. Human hepatocarcinoma cell line HepG2 and mouse hepatocarcinoma ascites cell line H-22 were provided by the Pharmacological Laboratory of the Pharmaceutical College, Zhejiang University. The transplanted S-180 sarcoma of mice was passaged in the laboratory and kept in ascites, according to the conventional method, and was subcultured every 4–5 days.

### Experimental Animals

Equal numbers of male and female, clean-grade ICR mice, weighing between 18 and 22 g at the time of inoculation, were purchased from the Animal Center of Zhejiang Academy of Medical Sciences with license number: SCXK (Zhejiang) 2008-0033. BALB/c-nude mice of SPF grade, 5–6 weeks old, were purchased from Hayslake Laboratory Animals Co. Ltd., with license number: SCXK (Shanghai) 2009-0005 and certificate number 0057827. All animal testing programs were submitted to the Zhejiang Chinese University Laboratory Animal Management Committee for review and approval. All the methods described here were approved and performed in accordance with the relevant guidelines and regulations.

### Preparation and Identification of PEC

#### Preparation of PEC

Briefly, 200.0 g of the *Polygonum perfoliatum L* component, extracted by ethyl acetate was separated by silica gel column chromatography, eluted with gradient elution using dichloromethane-methanol, and detected by thin layer chromatography (fluorescence or sulfuric acid-methanol coloration). Five different fractions were obtained at the sites of ethyl acetate-methanol (40:1), (35:1), (25:1), (15:1), and (5:1), which were recorded as PEA, PEB, PEC, PED, and PEE, respectively. After weighing, PEA was found to be 1.1 g (yield rate of 3.0%), PEB was 5.7 g (yield rate of 15.4%), PEC was 6.9 g (yield rate of 18.7%), PED was 8.0 g (yield rate of 21.7%), and PEE was 15.2 g (yield rate of 41.2%).

#### Composition Analysis of PEC

##### Reparation of sample solution

1.1 mg PEC was dissolved in methanol and prepared into 1.0 mg/mL sample solution for UHPLC-Q-TOF-MS analysis.

##### Chromatographic conditions

Chromatographic column: ACQUITY UPLC BEH C18 (150 × 2.1 mm, 1.7 μm). Mobile phase: 0.1% acetonitrile formate (A)-−0.1% water formate (B). Gradient elution procedure: 0~5 min 15~20% A; 5~32 min, 20~40% A; 32~37 min, 40~95% A. The volume of each injection was 4 μL and the volume flow rate was 0.3 mL/min.

##### Mass spectrum conditions

Time of Flight Mass Spectrometry uses TurboIon Spray ion source and ESI positive and negative ion scanning mode. The specific conditions were as follows: Ion Source Gas1(Gas1): 55, Ion Source Gas2(Gas2): 55, Curtain gas(CUR): 35, source temperature: 60°C, IonSapary Voltage Floating (ISVF): 5500 V/-4500 V; TOF MS scan m/z range: 50–1,500 Da, production scan m/z range: 50–1,000 Da, TOF MS scan accumulation time 0.25 s/spectra, product ion scan accumulation time 0.05 s/spectra; Secondary mass spectrometry uses IDA and high sensitivity mode, Declustering potential (DP): +60 V (positive and negative ion mode).

##### SCIEX OS software

SCIEX OS software contains multiple confidence criteria, including quality accuracy, retention time, isotopes, and matching use of compound libraries. In this experiment, TCM MS/MS Library (including secondary data of more than 1,500 Chinese herbal medicines) can be searched according to the first-order accurate mass number, isotope distribution ratio and MS/MS of the compounds, and then the screening of the target compounds can be completed.

### The Inhibitory Effect of PEC on Proliferation of Cancer Cells

#### The MTT Colorimetric Assay

Single cell suspension of tumor cells in logarithmic growth phase was seeded in 96-well plates at 100 μL/well. Thereafter, the plates were cultured in an incubator at 37°C, with 5% CO_2_ and saturated humidity for 24 h, following which, 5 groups of PEC with 1/2 decreasing ratio of concentration were added, respectively. According to the inhibitory effect of PEC on various cancer cells, the maximum concentration of PEC on various cancer cells was determined. Normal control group and blank group were set up, with 4 parallel wells in each group. The total well volume was 200 μL. After the plate was cultured in an incubator at 37°C, with 5% CO_2_ and saturated humidity, 20 μL MTT of 5 mg/mL were added to each well. After 4 h of incubation, the supernatant was discarded, dissolved in 150 μL/well DMSO, and shaken evenly. The OD value was measured at a wavelength of 570 nm using a microplate reader. The inhibition rate and IC_50_ of different concentrations of PEC on tumor cells was calculated. Each test was repeated 3 times.

#### Measurement of Cell Growth Curve

For the cell growth curve experiment, 100 μL of SGC-7901 and A549 cells in logarithmic growth phase were inoculated into 96-well plates with 1,500 and 2,000 cells, respectively, in each well. Following 24 h of incubation, 200 μL of PEC was added to each well and three concentration gradients (100, 50, 25 μg mL^−1^) were set. Each concentration was divided into 6 groups in parallel, while 6 sets were also prepared for the normal control group. Each group was provided with 4 parallel wells. After 0, 24, 48, 72, 96, and 120 h, 20 μL MTT of 5 mg/mL were added to each well. Thereafter, plates were incubated for further 4 h, after which, the supernatant from the well of the normal control group, as well as that from each of the concentration-administered groups, was discarded. The cells were then dissolved in 150 μL/well DMSO, and shaken evenly. The OD value was measured by a microplate reader at a wavelength of 570 nm, and the growth curve was plotted with time as the abscissa and OD as the ordinate.

### The Inhibitory Effect of PEC on Tumors in Animals

#### The Inhibitory Effect of PEC on Transplanted Tumor H22 in Miceon and Transplanted Tumor S-180 in Mice

After the tumor was inoculated in the abdomen, it was allowed to grow for 7–9 days, following which, ascetic fluid of H22 hepatoma mice were extracted under sterile condition after cutting off their necks. Thereafter, it was diluted with sterile saline to form a 1 × 10^7^ /mL tumor cell suspension. Under sterile conditions, 0.2 mL tumor cell suspension was inoculated subcutaneously in the right forelimb of equal numbers of male and female healthy mice. The day after inoculation, 10 mice in each group were randomly divided according to their body weight. High dose (14 mg/kg body weight), middle dose (7 mg/kg body weight), and low dose (3.5 mg/kg body weight) of PEC were administered orally to the respective groups once a day. The model group was administered the same volume of normal saline for 8 consecutive days. The positive drug (Cytoxan, CTX) control group was intraperitoneally injected with cyclophosphamide (25 mg/kg/day). On the 9th day after the administration, the H22 liver cancer mice were sacrificed by cervical dislocation. The tumor masses were completely stripped and weighed. The tumor inhibition rate was calculated by the following formula. The experiment was repeated in three batches, each with equal numbers of male and female mice. The transplanted tumor S-180 in mice experiment was conducted in the same way as H22 hepatocellular carcinoma.

#### The Effect of PEC on Human Gastric Tumor SGC-7901 Transplanted in Nude Mice

SGC-7901 cells in their logarithmic growth phase (cultured *in vitro*) were trypsinized, collected, and centrifuged. PBS was used to prepare a cell suspension with a concentration of 1 × 10^7^ cells/mL. Tumor cell suspension (0.2 mL) was inoculated subcutaneously on the right side of the back of five nude mice. When the tumor grew to about 1,000 mm^3^, the nude mice were sacrificed, and the tumor was removed, cut into 5 mm^3^ pieces with surgical scissors, and inoculated into the right back of the nude mice. After the tumor was passaged for three generations following this method, the third-generation tumor masses were inoculated in 50 mice. Tumor conditions and tumor size were observed. When the tumor volume was 100–300 mm^3^, 30 nude mice with similar tumor volume and good shape were selected and divided into five groups, with six mice in each group: model group, PEC (3.5, 7, and 14 mg/kg), which received intragastric administration once a day. The control group was administered the same volume of normal saline for 8 consecutive days. The positive drug (CTX) group was intraperitoneally injected with cyclophosphamide (25 mg/kg/day). Tumor volume (TV) and body weight were measured three times per week, for 30 consecutive days. The nude mice were sacrificed after they had been kept fasting for 14 h, following which, the tumor masses were stripped. The relative tumor volume (RTV) and the relative tumor growth rate (T/C%) were calculated. The experiment was repeated three times.

$$\begin{array}{l} \text{​​​​ TV}=1/2\times {\text{D}}_{1}\times \;{\text{D}}_{2}^{2}\\ \text{ RTV}={\text{V}}_{\text{t}}{\text{/V}}_{0}\\ \text{T/C}\%={\text{T}}_{\text{RTV}}{{\text{/C}}_{\text{RTV}}}^{*}100\% \end{array}$$

In the formula, D_1_ and D_2_ represent the length and width of the tumor, respectively. V_0_ is the tumor volume at the time of administration according to the different cages and V_t_ is the tumor volume at each measurement. T_RTV_ is the RTV of administration groups and C_RTV_ is the RTV of model group.

### Anti-tumor Mechanism of PEC

#### Detection of Apoptosis by Fluorescent Staining

A single-cell suspension of SGC-7901 cells in logarithmic growth phase was prepared, and the cell concentration was adjusted to 1 × 10^4^ cells, which was inoculated in a 6-well culture plate, with 2 mL per well; one well was designated as the normal control group. After the plate was cultured in an incubator for 24 h at 37°C, with 5% CO_2_ and saturated humidity, 2 mL of PEC at concentrations of 50, 25, and 12.5 μg/mL, respectively, were added to the wells designated as administration groups. An equal amount of culture medium was added to the normal control group, and placed in an incubator at 37°C, 5% CO_2_, and saturated humidity for 48 h. The morphology of SGC-7901 tumor cells was observed using an inverted microscope, and the difference between the normal control group and each administration group was compared. To each well, 5 μL acridine orange was added at room temperature and incubated away from light for 10 min, followed by 3 washes with PBS. The morphology of the cells was observed under a fluorescence microscope, and the difference between the normal control group and each administration group was compared.

#### Detection of Apoptotic Rate by Flow Cytometry

SGC-7901 single cell suspension, in logarithmic growth phase, was prepared and inoculated into 100 mL culture flask, after adjusting cell density to 5 × 10^5^/bottle. PEC was added to the administration groups to achieve final concentrations of 50, 25, and 12.5 μg/mL, respectively. An equal amount of culture medium was added to the normal control group and cultured for 24 h. Thereafter, the cells were centrifuged at 1,000 rpm for 5 min, and washed twice with PBS to remove cell debris from the cell suspension. Approximately, 1~5 × 10^5^ cells were collected, to which 500 μL annexin-binding buffer was added evenly by blowing. Thereafter, 5 μL annexin V-FITC and 10 μL PI were added and incubated for 10 min away from light. The percentage of apoptotic cells was measured by flow cytometry. Each test was repeated 3 times.

#### Effect of PEC on Cell Cycle Progression of SGC-7901 Tumor Cells

SGC-7901 single-cell suspension, in logarithmic growth phase, was inoculated into 100 mL culture flask, by adjusting cell density to 5 × 10^5^/bottle. PEC was added to the administration groups to achieve a final concentration of 50, 25, and 12.5 μg/mL, respectively. An equal volume of culture medium was added to the normal control group and cultured for 24 h. Thereafter, the cells were centrifuged at 1,000 rpm for 5 min, and washed twice with PBS to remove cell debris from the cell suspension. Approximately, 1~5 × 10^5^ cells were collected, fixed in 70% ethanol, and stored at 4°C. Thereafter, the fixative solution was washed away with PBS, followed by addition of 100 mL RNase A. The cells were then incubated for 30 min in a 37°C water bath; 400 mL PI was used for dyeing and mixing. The mixture was detected by FCM after incubating for 30 min at 4°C, away from light. Cells were collected by 630 band-pass filters through FSC/SSC scatter plots. Adhesive cells and debris were excluded by gate-setting technique. The percentage of cell cycle on fluorescence histogram was analyzed.

### The Inhibitory Effect of PEC on Angiogenesis of Tumors

#### Detection of Tumor Necrosis by HE Staining

After dissection of the tumor, it was fixed with 10% neutral buffered formalin. The specimen was embedded in conventional paraffin, and made into 4 μm tissue sections, dewaxed by xylene, dehydrated by gradient alcohol, and washed twice with distilled water, each time for 3 min. Thereafter, the specimen was stained with Gill hematoxylin for 10 min, washed with distilled water for 2 min, and observed under a microscope. If deemed necessary, the specimen was differentiated by 0.5% ethanol hydrochloride for several seconds, washed in flowing water, blued in warm water, stained by 95% ethanol for 1 min, followed by 0.5% ethanol staining solution for 1 min, and dehydrated by gradient alcohol twice, each time for 3 min. Xylene was used twice (5 min, each time) for transparency and neutral gum was used for sealing. The results were observed under ordinary optical microscope.

#### Immunohistochemical Staining for the Measurement of MVD and VEGF Expression

The MVD strength and expression of VEGF was detected by immunohistochemical staining. MVD positive intensity was based on the brown staining of CD31 reaction in vascular endothelial cells. First, the whole section was observed under the low power lens of the microscope (×100) to determine the highest vascular density in the tumor, and 5 areas with the largest number of microvessels were selected. Thereafter, the number of blood vessels in these five fields was recorded after observing under the high power lens of the microscope (×200), and the average number was taken as the MVD of the case. For determining VEGF positivity, the following criteria was set: first, the intensity of staining was graded; the depth of staining was compared with the background. Points were assigned based on the staining intensity: 0 points for colorless, 1 point for light yellow, 2 points for brownish yellow, and 3 points for brown. Subsequently, the percentage of positive cells was scored: 0 was negative, positive cells <10% were assigned 1 point, 11–50% were assigned 2 points, 51–75% were assigned 3 points, and >75% were assigned 4 points.

### The Effect of PEC on Immune Function of S-180 Sarcoma Mice

#### The Effect on the Proliferation of Splenic Lymphocytes in S-180 Sarcoma Mice

After 24 h of the last administration, the spleens of the mice were collected under aseptic conditions, and the spleen cells were extracted; appropriate amount of RPMI 1640 medium was used for preparation of the cell suspension. The cells were counted by 0.4% trypan blue exclusion method and were diluted with RPMI1640 medium. A 96-well plate was taken, and 100 μL spleen cell suspension was added parallelly to 12 wells. Thereafter, 100 μL RPMI 1640 medium, 100 μL Concanavalin A (ConA) dilution solution and 100 μL LPS dilution solution were added and 4 wells were set in parallel. They were incubated for 44 h at 37°C with 5% CO_2_; 20 μL MTT solution (5 mg/mL) was added to each well and incubated for 4 h. After centrifugation at 1,800 rpm for 5 min, the supernatant was discarded and 150 μL DMSO (containing 4% 1 N hydrochloric acid) was added to each well to dissolve the crystals by oscillation, away from light. OD value was measured at 570 nm using a microplate reader, and stimulation index (SI) was calculated as follows: SI = OD value of the original culture with mitogen/OD value of the original culture without mitogen.

### Determination of Killing Activity of Natural Killer Cells (NK) and Cytotoxic T Lymphocytes (CTL) in Mice

#### Preparation of Target Cells

Human chronic myeloid leukemia K562 cells and S180 cells were collected from the exponential growth phase, and RPMI 1640 medium was added to prepare a cell suspension with a concentration of 2.0 × 10^5^/mL.

#### Preparation of Effector Cells

The cell concentration of the splenocyte suspension prepared above was 1.0 × 10^7^/mL.

#### Cytotoxicity Assay

RPMI 1640 culture medium was added to each well in columns 1–4 of a 96-well cell culture plate. Subsequently, K562 cell suspension was added to each well in columns 5–8, and S-180 cell suspension was added to each well in columns 9–12. Spleen cell suspension (100 μL) was added to B to G lines, and 100 μL RPMI 1640 medium was added to A and H lines. After the plate was cultured at 37°C for 24 h in a 5% CO_2_ incubator, 20 μL MTT solution (5 mg/mL) was added to each well 4 h before the end of the incubation, after which, incubation was continued for completion of 24 h. After centrifugation at 1,800 rpm for 5 min, the supernatant was discarded. Thereafter, 150 μL DMSO, containing 4% 1 N hydrochloric acid, was added to each well to dissolve the crystals by oscillation away from light. OD value was measured at 570 nm to calculate NK cell activity and CTL activity.

### Determination of Ability of Spleen Cells to Secrete Interleukin 2 (IL-2)

#### IL-2 Induction of Splenocytes and Preparation of Culture Supernatant

The spleen cell suspension prepared above was inoculated into a 24-well cell culture plate, and 1 mL spleen cell suspension and an equal volume of ConA solution were added to each well to achieve a final concentration of 10 μg/mL. To the control group, 1 mL RPMI 1640 medium was added. The plate was incubated at 37°C for 48 h in a 5% CO_2_ incubator, after which cells were centrifuged at 2,000 rpm for 10 min. Thereafter, the supernatant was filtered through a 0.45 μm microporous membrane. The filtrate was collected and stored at −20°C, which contained the IL-2 to be tested.

#### Preparation of Target Cells

Normal ICR mice were sacrificed by cervical dislocation. Spleen cell suspension was prepared according to the conventional method, and the cell concentration was adjusted to 5 × 10^6^/mL by RPMI1640 complete culture medium. An equal volume of ConA solution, with final concentration of 5 μg/mL, was added and the suspension was cultured at 37°C for 48 h in a 5% CO_2_ incubator. It was washed twice with PBS, and RPMI 1640 medium was added to adjust the cell concentration to 5 × 10^6^/mL, which served as the target cells for IL-2 assay.

#### Sample Testing

The supernatants of IL-2 from each group preserved at 20°C were assayed for their IL-2 levels using ConA-activated splenic lymphocytes of mice. In a 96-well cell culture plate, 100 μL target cell suspension and 100 μL supernatant dilution (1:4) were added to each well, and 4 parallel wells were set for each sample. At the same time, a sample containing the corresponding concentration of ConA and the culture solution was prepared. They were incubated at 37°C for 20 h, in a 5% CO_2_ incubator, after which, 20 μL MTT solution (5 mg/mL) was added to each well. Thereafter, incubation was continued for another 4 h, after which, samples were centrifuged at 1,800 rpm for 5 min; the supernatant was discarded and 150 μL DMSO containing 4% 1 N hydrochloric acid was added to each well to dissolve the crystals by oscillation away from light. They were placed in the dark at room temperature for 15 min, and the OD value was measured at a wavelength of 570 nm using a microplate reader. The proliferation index (SI) was calculated as follows.

#### Detection of Specific Antibodies and Subclasses of Serum Antigens

On a 96-well microtiter plate, 100 μL coating solution (50 mM carbonate buffer, S180 cell lysate with protein concentration of 50 g/mL, pH 9.6) was added to each well which was then cultured at 4°C for 24 h. Thereafter, the plate was washed with washing solution 3 times, for 3 min each. Blocking solution (150 μL) was added to each well, and the plate was incubated at 37°C for 2 h in a 5% CO _2_ incubator. The plate was then washed 3 times with washing solution, 3 min for each, after which, 100 μL (1:200) serum dilutions were added to each well. Plates were incubated at 37°C for 2 h in a 5% CO _2_ incubator, followed by 3 washes of 3 min each, with washing solution. Thereafter, 100 μL horseradish peroxidase-labeled rabbit anti-mouse IgG (1:16,000), goat anti-mouse IgG2a (1:8,000), and goat anti-mouse IgG2b (1:8,000) antibody dilutions were added to each well. The plate was then incubated at 37°C for 2 h in a 5% CO _2_ incubator, followed by 3 washes of 3 min each with washing solution. Thereafter, 100 μL substrate solution was added to each well. The plate was placed in the dark at 37°C for 10 min, followed by addition of NH_2_SO_4_ solution to terminate the reaction. The OD value was measured at a wavelength of 492 nm using a microplate reader.

## Results

### Composition Analysis of PEC

**Figure 1:**
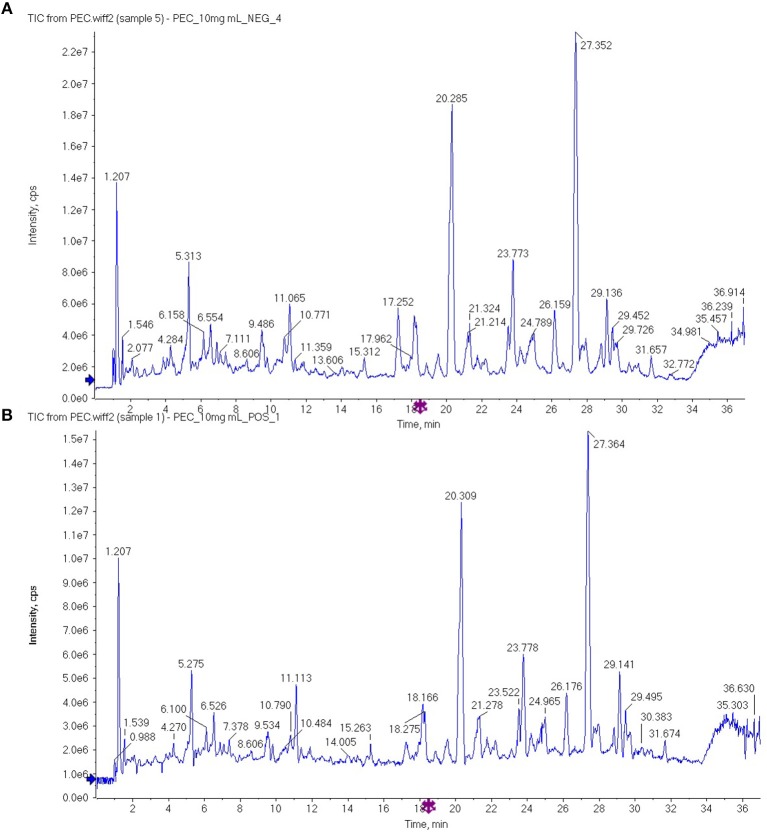
The total ion flow diagram of PEC by UHPLC-Q/TOF-MS in **(A)** negative ion mode and **(B)** positive ion mode.

The PEC sample solution was analyzed by UHPLC-Q/TOF-MS system, and the total ion flow diagram of PEC was obtained. By comparing and screening with TCM MS/MS Library in SCIEX OS software, the compounds were identified qualitatively. The identification results are shown in Figure 1 and Tables 1, 2. Thirty one compounds were identified under negative ion mode and 47 compounds under positive ion mode. Most of these compounds were the flavonoids, and it can be inferred that the main anticancer component in PEC are flavonoids.

**Table 1:** High resolution mass spectrometry data and elemental composition of PEC (Negative Ion Mode).

**No**	**Retention time**	**Found at mass**	**Precursor mass**	**Mass error (ppm)**	**Formula**	**Library hit**
1	5.31	463.0879	463.088	−0.7	C_21_H_20_O_12_	Isoquercitrin
2	5.31	463.0879	463.088	−0.7	C_21_H_20_O_12_	Hyperin
3	1.55	169.014	169.014	−1.6	C_7_H_6_O_5_	Gallic acid
4	6.95	447.0929	447.093	−0.9	C_21_H_20_O_11_	Astragalin
5	4.5	300.9987	300.999	−0.8	C_14_H_6_O_8_	Ellagic acid
6	14.02	593.1295	593.13	−0.9	C_30_H_26_O_13_	Tiliroside
7	2.09	179.035	179.035	0.2	C_9_H_8_O_4_	Caffeic acid
8	9.42	435.1293	435.13	−0.8	C_21_H_24_O_10_	Phloridzin
9	8.49	359.0772	359.077	−0.1	C_18_H_16_O_8_	Rosmarinic acid
10	12.68	301.0354	301.035	−0.1	C_15_H_10_O_7_	Quercetin
11	9.36	431.0983	431.098	−0.2	C_21_H_20_O_10_	Afzelin
12	5.22	441.0831	441.083	1	C_22_H_18_O_10_	Catechin Gallate
13	9.12	463.0882	463.088	−0.1	C_21_H_20_O_12_	Quercetin-3′-O-glucoside
14	2.8	179.0349	179.035	−0.7	C_9_H_8_O_4_	Caffeic acid
15	35.46	795.4534	795.454	−0.3	C_41_H_66_O_12_.HCOOH	α-Hederin +HCOOH
16	22.04	695.4013	695.401	0.2	C_36_H_58_O_10_.HCOOH	Pedunculoside +HCOOH
17	15.32	593.1302	593.13	0.2	C_30_H_26_O_13_	Tiliroside
18	2.68	137.0244	137.024	0.1	C_7_H_6_O_3_	Protocatechuic Aldehyde
19	12.49	461.109	461.109	0.1	C_22_H_22_O_11_	Pratensein-7-O-glucoside
20	4.07	153.0193	153.019	0	C_7_H_6_O_4_	Protocatechuic acid
21	1.95	153.0192	153.019	−0.9	C_7_H_6_O_4_	Protocatechuic acid
22	8.41	461.1088	461.109	−0.4	C_22_H_22_O_11_	Tectoridin
23	11.39	415.1037	415.103	0.6	C_21_H_20_O_9_	Puerarin
24	2.93	177.0195	177.019	0.8	C_9_H_6_O_4_	Daphnetin
25	2.93	177.0195	177.019	0.8	C_9_H_6_O_4_	Esculetin
26	22.67	695.4017	695.401	0.8	C_36_H_58_O_10_.HCOOH	Pedunculoside +HCOOH
27	6.01	525.3074	525.307	1	C_27_H_44_O_7_.HCOOH	β-Hydroxyecdysone +HCOOH
28	6.01	525.3074	525.307	1	C_27_H_44_O_7_.HCOOH	β-Ecdysone +HCOOH
29	1.13	134.0474	134.047	1	C_5_H_5_N_5_	Adenine
30	14.06	283.061	283.061	−0.7	C_16_H_12_O_5_	Wogonin
31	6.7	525.3073	525.307	0.7	C_27_H_44_O_7_.HCOOH	Epibrassinolide +HCOOH

**Table 2:** High resolution mass spectrometry data and elemental composition of PEC (Positive Ion Mode).

**No**	**Retention time**	**Found at mass**	**Precursor mass**	**Mass error (ppm)**	**Formula**	**Library hit**
1	5.28	465.1031	465.103	0.6	C_21_H_20_O_12_	Isoquercitrin
2	5.28	465.1031	465.103	0.6	C_21_H_20_O_12_	Hyperin
3	5.28	303.0502	303.05	0.8	C_15_H_10_O_7_	Morin hydrate
4	5.28	303.0502	303.05	0.8	C_15_H_10_O_7_	Quercetin
5	6.55	303.0498	303.05	−0.4	C_15_H_10_O_7_	Morin hydrate
6	6.55	303.0498	303.05	−0.4	C_15_H_10_O_7_	Quercetin
7	7.08	303.0497	303.05	−0.8	C_15_H_10_O_7_	Quercetin
8	6.9	449.1081	449.108	0.6	C_21_H_20_O_11_	Luteoloside
9	6.9	449.1081	449.108	0.6	C_21_H_20_O_11_	Cyanidin-3-O-glucoside
10	6.9	449.1081	449.108	0.6	C_21_H_20_O_11_	Astragalin
11	7.39	317.0657	317.066	0.3	C_16_H_12_O_7_	Isorhamnetin
12	4.92	433.1128	433.113	−0.3	C_21_H_20_O_10_	Isovitexin
13	9.61	303.0506	303.05	2.1	C_15_H_10_O_7_	Morin hydrate
14	9.61	303.0506	303.05	2.1	C_15_H_10_O_7_	Quercetin
15	14.08	593.1862	593.186	−0.4	C_28_H_32_O_14_	Linarin
16	6.9	287.0554	287.055	1.3	C_15_H_10_O_6_	Luteolin
17	6.9	287.0554	287.055	1.3	C_15_H_10_O_6_	Scutellarein
18	5.6	449.1082	449.108	0.8	C_21_H_20_O_11_	Luteoloside
19	5.6	449.1082	449.108	0.8	C_21_H_20_O_11_	Cyanidin-3-O-glucoside
20	5.6	449.1082	449.108	0.8	C_21_H_20_O_11_	Astragalin
21	7.08	449.1082	449.108	0.9	C_21_H_20_O_11_	Quercitrin
22	7.08	449.1082	449.108	0.9	C_21_H_20_O_11_	Quercetin 7-rhamnoside; Vincetoxicoside B
23	7.08	449.1082	449.108	0.9	C_21_H_20_O_11_	Rhodionin
24	14	595.1458	595.145	1.9	C_30_H_26_O_13_	Tiliroside
25	8.4	463.1246	463.123	2.3	C_22_H_22_O_11_	Pratensein-7-O-glucoside
26	12.51	463.1248	463.123	2.8	C_22_H_22_O_11_	Pratensein-7-O-glucoside
27	1.14	136.0615	136.062	−1.9	C_5_H_5N5_	Adenine
28	9.38	287.0556	287.055	2.2	C_15_H_10_O_6_	Luteolin
29	5.19	443.0982	443.097	2.2	C_22_H_18_O_10_	Catechin Gallate
30	5.19	443.0982	443.097	2.2	C_22_H_18_O_10_	Epicatechin Gallate
31	5.76	625.1763	625.176	0	C_28_H_32_O_16_	Neferine
32	5.83	433.1138	433.113	2.1	C_21_H_20_O_10_	Apigenin-7-glucoside
33	5.83	433.1138	433.113	2.1	C_21_H_20_O_10_	Sophoricoside
34	5.83	433.1138	433.113	2.1	C_21_H_20_O_10_	Genistin
35	3.85	449.1096	449.108	3.9	C_21_H_20_O_11_	Homoorientin \
36	3.85	449.1096	449.108	3.9	C_21_H_20_O_11_	Orientin
37	7.72	417.1197	417.118	4	C_21_H_20_O_9_	Puerarin
38	35.46	455.3534	455.352	3.1	C_30_H_46_O_3_	Betulonic acid
39	4.89	319.0458	319.045	3.1	C_15_H_10_O_8_	Myricetin
40	7.56	354.1346	354.134	2.9	C_20_H_19_NO_5_	Protopine
41	22.05	489.3593	489.357	3.7	C_30_H_48_O_5_	Asiatic acid
42	1.15	118.0862	118.086	−0.3	C_5_H_11_NO_2_	Betaine
43	1.16	268.1041	268.104	0.4	C_10_H_13_N_5_O_4_	Adenosine
44	8.55	449.1456	449.144	3	C_22_H_24_O_10_	D Licoagroside D
45	5.97	481.3172	481.316	2.6	C_27_H_44_O_7_	β-Ecdysone
46	3.37	355.1038	355.102	4	C_16_H_18_O_9_	Scopolin
47	22.05	668.4394	668.437	3.8	C_36_H_58_O_10_.NH3	Pedunculoside +NH3

### The Broad-Spectrum Cytotoxicity of PEC Against Various Cancer Cell Lines

The MTT assay indicated that PEC exerted cytotoxicity against various cancer cell lines. At the maximum concentration of 100 or 120 μg/mL, PEC showed an inhibition ratio of over 70% in all cancer cell lines. Among them, SGC-7901, PC-3, and PANC-1 were the most sensitive cell lines with IC_50_ values from 20.6 to 26.2 μg/mL. The other cell lines showed less sensitivities with IC_50_ values > 40 μg/mL (Table 3).

**Table 3:** Inhibitory effect of PEC on cancer cells after 72 h ($\bar{x}$ ± s, *n* = 4).

**Cancer cell lines**	**PEC (μg·mL^**−1**^)**	**IC_**50**_ (μg·mL^**−1**^)**	**Inhibition rate (%)**
Hela	100	40.0 ± 1.8	82.4 ± 0.6
SGC-7901	100	26.2 ± 2.2	88.7 ± 0.5
PC-3	100	25.5 ± 2.6	88.6 ± 0.2
A549	120	47.7 ± 2.3	72.0 ± 0.8
PANC-1	100	20.6 ± 2.4	89.7 ± 0.4
HepG2	120	41.0 ± 2.1	70.1 ± 0.7
BT-325	120	43.1 ± 2.4	75.9 ± 0.3

### The Inhibitory Effects of PEC on Cancer Cell Growth

**Figure 2:**
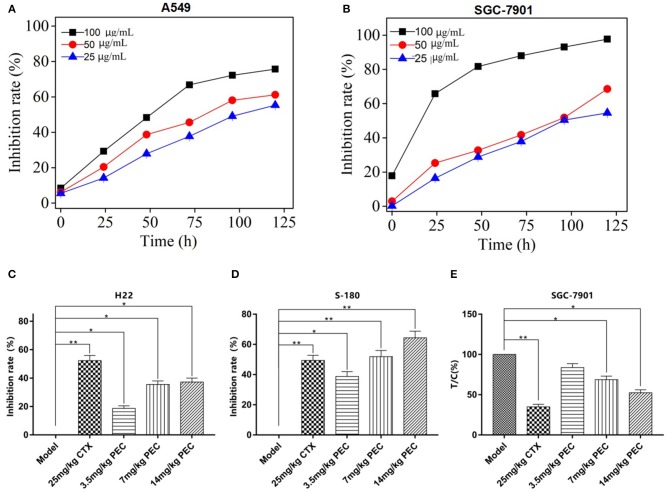
Inhibitory effects of PEC on growth of **(A)** SGC-7901 and **(B)** A549 cells with different time ($\bar{x}$ ± s, *n* = 4). Inhibitory effects of PEC on **(C)** H22 and **(D)** S-180 xenograft tumors in mice ($\bar{x}$ ± s, *n* = 10). **(E)** Inhibitory effects of PEC on transplanted SGC-7901 tumors in nude mice ($\bar{x}$ ± s, *n* = 6). Compared with model group: *P < 0.05, **P < 0.01. Tumor inhibition rate (%) = (mean tumor weight in the model group – mean tumor weight in the administration group)/average tumor weight in the model group × 100%.

To further demonstrate the anticancer activity of PEC, one sensitive cell line SGC-7901 and one cell line with less sensitivity A549 were selected for further investigation for their growth curves under PEC treatment. As shown in Figure 2, the inhibitory effect of PEC on the two cancer cell lines constantly strengthened in a time-dependent manner. At the same time point, the inhibition ratios became higher along with an increase in PEC concentration, indicating a strong dose-effect relationship (Figures 2A,B). In addition, with the same dose and at the same time point, the inhibition ratio of SGC-7901 was significantly higher than that of A549, confirming that the SGC-7901 cells were more sensitive to PEC.

### The Inhibitory Effects of PEC on Tumor Growth *in vivo*

We further determine the *in vivo* efficacy of PEC in different xenograft models. As shown in Figures 2C,D, the dose-dependent inhibitory effects were observed on the growth of H22 and S-180 tumors in mice administrated with PEC. At the dose was 14 mg/kg, the inhibitory rates were 37.25 and 60.0%, respectively, indicating a good dose-effect relation with a significant difference compared to the model group (*P* < 0.05).

PEC also exerted an inhibitory effect on the growth of transplanted SGC-7901 tumor in nude mice. At the dose of 14 mg/kg, PEC inhibited 53% tumor growth, with a significant difference compared to the model group (*P* < 0.05; Figure 2E). In addition, the PEC treatment did not make any changes in the mouse body weights in comparison to the control group, which indicated that PEC did not cause any host toxicity *in vivo* (S-Tables 1–3). However, CTX (cytoxan) treatment showed significant effects on the average body weights of mice, which might be related to its immunosuppressive and toxic effects.

### Anti-tumor Mechanism of PEC

#### PEC Induced Cancer Cell Apoptosis

**Figure 3:**
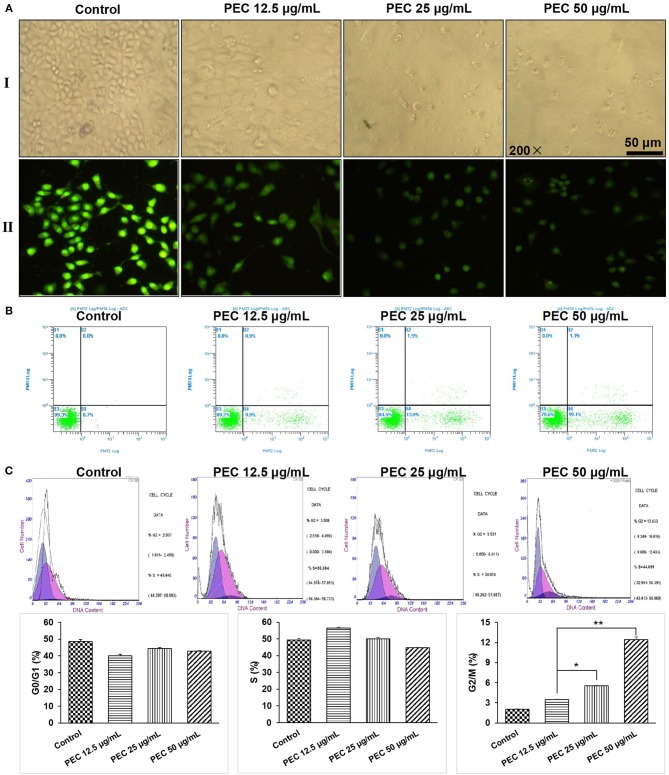
Effects of PEC on the morphology of SGC-7901 cells in **(AI)** the inverted microscope and **(AII)** the fluorescent microscope. **(B)** Detection of apoptosis of SGC-7901 induced by PEC in FCM. **(C)** Effect of PEC on SGC-7901 in Periodic Blockade. Compared with control group: *P < 0.05, **P < 0.01.

We further determined the mechanisms for PEC's anticancer activity. As shown in Figure 3A, under an inverted microscope, the SGC-7901 cells belonging to the normal control group appeared plump, thriving and stereoscopic, adhering to the wall, with a single shape, and high refractive index. However, along with an increasing concentration of PEC, the cell number decreased, the shape of the cells appeared irregular and shrunk, the number of smudged cells increased, and the refractive index decreased. At the concentration of 50 μg/mL, the intensity of cells decreased drastically, and all the cells appeared disrupted. When observed under a fluorescent microscope, SGC-7901 cells belonging to the normal control group appeared to have larger nuclei, even nuclear chromatins with green fluorescence, and active cell division. With increasing concentration of PEC, features indicative of apoptosis became evident, including karyopyknosis, nuclear fragmentation, partial nucleus fragmentation and uneven nuclear chromatins, with densely stained yellow and green fluorescence. At the concentration of 50 μg/mL, obvious nucleus fragmentation, chromatin shrinkage, red dead cells, orange terminal apoptotic cells, and apoptotic bodies were visible. These results together indicated that PEC induced apoptosis of SGC-7901 cells in a concentration-dependent manner.

We further determined the apoptotic rates of SGC-7901 cells by flow cytometry (Figure 3B). The apoptotic rate in the normal control group was found to be 0.7%, while early apoptotic cells and apoptosis rate was found to increase with increasing PEC dosage. When treated with 50 μg/mL PEC, the apoptotic rate increased from 0.7 to 19.1%, with significant difference compared to the normal control group (*P* < 0.01), confirming that PEC induced apoptosis of SGC-7901 cells in a dose-dependent manner.

#### PEC Arrested Cancer Cells at G2 Phase

The effects of PEC on cell cycle progression were determined. As shown in Figure 3C, in the control group, cells in G1, S, and G2/M phase were 48.53, 49.44, 2.03%, respectively. Under the treatment of PEC, the proportion of cells in the G1 phase gradually decreased, while the proportion of cells in the G2 phase gradually increased. When the concentration of PEC reached 50 μg/mL, cells at G2/M phase increased to 12.43%. However, the proportion of cells in the S phase remained unchanged. These results indicated that PEC could act on the G2/M level of the SGC-7901cells.

### The Inhibitory Effects of PEC on Angiogenesis of Tumors

**Figure 4:**
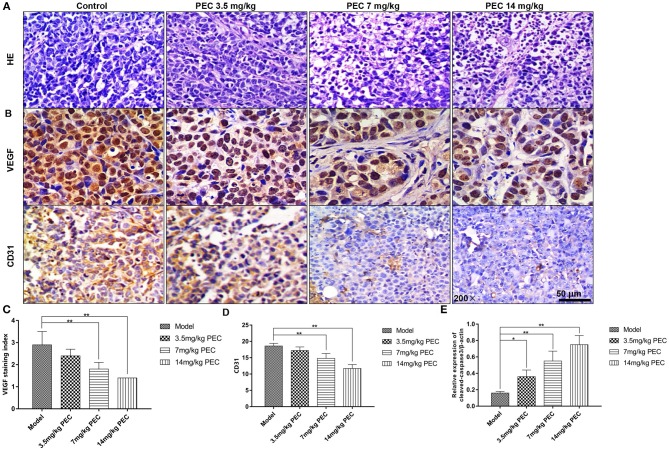
Effects of PEC on tumor tissues (HE × 200) **(A)** and VEGF **(B,C)** and CD31 **(B,D)** expression in SGC-7901 tumor tissue (IHC × 200). The expression of cleaved-casease3/β-actin in SGC-7901 tumor tissue **(E)**. Compared with model group: *P < 0.05, **P < 0.01.

As shown in Figure 4A, HE-stained tumor cells from the normal control group, viewed under an optical microscope, appeared to have plump nuclei and were stained uniformly blue. However, with increasing doses of PEC, the extracellular matrix in the PEC treated group appeared solidified, and the unstructured areas of red staining gradually increased. Nuclear fragmentation and dissolution were observed in tumor tissues from the group that had been administered the highest dose of PEC. Some necrotic tissues showed homogeneous red staining without structural areas, and the necrotic foci were significantly more than that observed in the normal control group. The above phenomenon indicated that PEC could promote tumor tissue necrosis in a dose-dependent manner.

### Immunohistochemical Staining for the Measurement of VEGF and CD31 Expression

As shown in Figures 4B,C, immunohistochemical analyses indicated that most cells in the normal control group were stained claybank, with some cell sepia showing strong VEGF expression. In the PEC-treated groups, a dose-dependent decrease was observed in the number of positive cells, indicating the inhibition of VEGF expression *in vivo*. In addition, the control group was also found to have abundant microvessels, with the nuclei of the vascular endothelial cells stained uniformly yellow, and the endothelial cells closely connected (Figures 4B,D). However, in the PEC-treated tumors, endothelial cells, and CD31-positive intensity significantly decreased. In the highest dose group, there were fewer positive vascular endothelial cells, which was significantly different from the control group (*P* < 0.01). These results indicated that PEC exerted inhibitory effects on tumor blood vessels in a dose-dependent manner. Moreover, we also examined the apoptosis level by Western blotting analysis of cleaved-caspase3. As shown in Figure 4E, PEC treatment significantly increased the expression of cleaved-caspase-3 in tumor tissues of mice.

### The Effect of PEC on Immune Function of S-180 Sarcoma Mice

#### The Effect on the Proliferation of Splenic Lymphocyte in S-180 Sarcoma Mice

**Figure 5:**
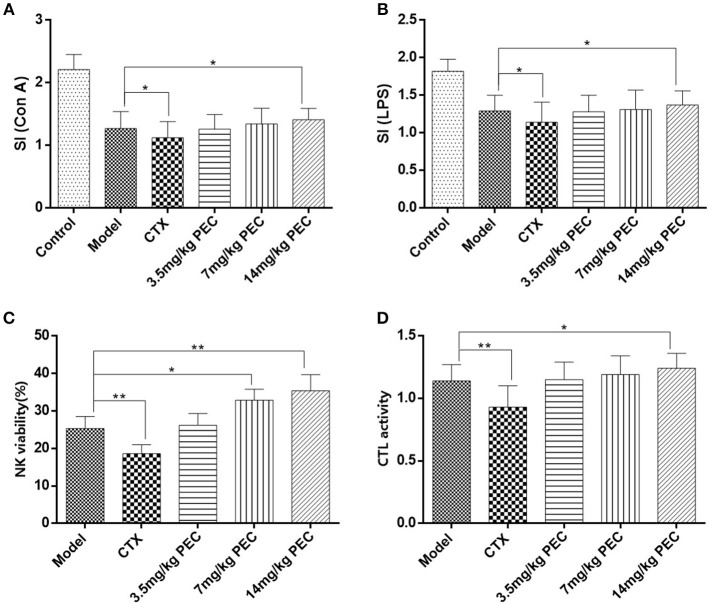
Effects of PEC on splenic lymphocyte proliferation induced by **(A)** ConA and **(B)** LPS in S-180 tumor-bearing mice. **(C)** Effects of PEC on NK in S-180 tumor-bearing mice ($\bar{x}$ ± s, *n* = 10). **(D)** Effects of PEC on CTL in S-180 tumor-bearing mice ($\bar{x}$ ± s, *n* = 10). Compared with model group: *P < 0.05, **P < 0.01.

We first studied the effect of PEC on the proliferation response of S-180 sarcoma mice that were induced by Con A and LPS. As shown in Figures 5A,B, compared to the normal mice, the proliferation capacity of T and B lymphocytes in the tumor-bearing mice was significantly reduced (*P* < 0.001). PEC treatment dose-dependently enhanced the proliferation capacity of T and B lymphocytes in mice (*P* < 0.05). However, T and B lymphocytes were found to be significantly reduced in the CTX control group (*P* < 0.05).

#### Killing Activity of Natural Killer Cells (NK) and Cytotoxic T Lymphocytes (CTL) in Mice

The effects of PEC on the activity of NK cells (Figure 5C) and CTL (Figure 5D) was also studied in S-180 tumor-bearing mice. The highest dose of PEC could significantly improve the S-180 tumor-burdened NK cell activity (*P* < 0.01) as well as the activity of CTL (*P* < 0.05) dose-dependently. In the CTX control group, NK cells and CTL activity were significantly lower than the tumor-bearing control group (*P* < 0.01).

#### The Effect of PEC on IL-2 Production in Spleen Cells of S-180 Tumor-Bearing Mice

**Figure 6:**
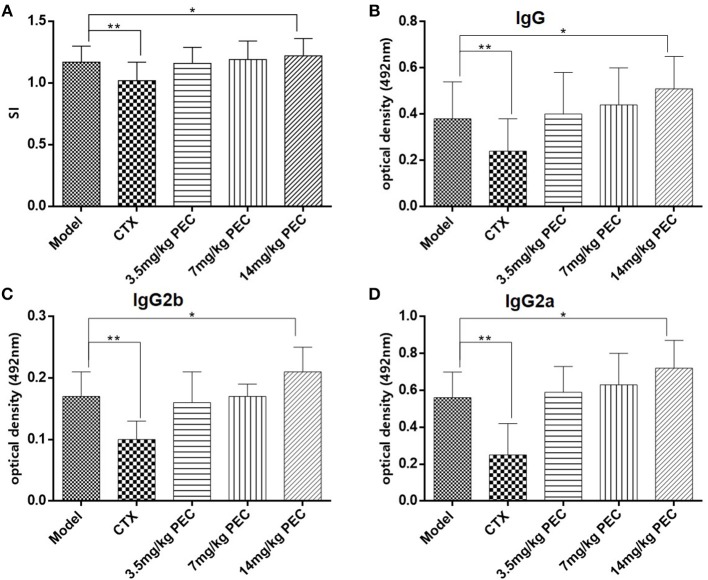
**(A)** Effects of PEC on IL-2 production by splenic lymphocytes in S-180 tumor-bearing mice ($\bar{x}$ ± s, *n* = 10). Effects of PEC on **(B)** IgG, **(C)** IgG2b, and **(D)** IgG2a in Serum of S-180 tumor-bearing mice ($\bar{x}$ ± s, *n* = 10). Compared with model group: *P < 0.05, **P < 0.01.

The effect of PEC on IL-2 production in spleen cells of S-180 sarcoma mice was studied (Figure 6A). It was observed that IL-2 production by the spleen cells of mice belonging to the CTX control group was significantly lower than that of the model group mice (*P* < 0.01). In the PEC groups, IL2 production increased with the increasing doses of PEC, showing a dose-effect relationship.

#### Detection of Specific Antibodies and Subclasses of Serum Antigens

The effect of PEC on specific antibodies and their subclasses in the serum of S-180 tumor-bearing mice were studied (Figures 6B–D). Significantly reduced levels of tumor antigen-specific IgG, IgG2a, and IgG2b were observed in the serum of tumor-bearing mice in the CTX group (*P* < 0.01), while increased levels were observed in the PEC group. This observation suggested that PEC mainly stimulated the Th1 type of immune response *in vivo*.

## Discussion

According to the clinical experience and previous studies of *P. perfoliatum* L. an original plant of PEC in Chinese folk, we have chosen seven cancer cell lines, including a human cervical cancer line (Hela), a human gastric cancer cell line (SGC-7901), a human prostate cancer cell line (PC-3), a human lung cancer cell line (A549), a human glioma cell line (BT-325), a human pancreatic cancer cell line (PANC-1), and a human hepatoma cell line (HepG2) to investigate the anticancer activity of PEC *in vitro*. An MTT assay was used to investigate the inhibitory effects of PEC on the proliferation of various tumor cells. The results showed that PEC could inhibit the growth of all cell lines. SGC-7901, PC-3, and PANC-1 cells were found to be more sensitive to PEC than Hela, A549, BT-325, and HepG2 cells. At the same time, it was found that PEC showed a significant dose-effect relationship in the inhibition of the tumor cells. We, therefore, selected SGC-7901 cells that were sensitive to PEC and A549 cells that were less sensitive to PEC to perform the concentration- and time-dependent studies (Figures 2A,B). The results showed that PEC had an obvious dose-effect relationship and time-effect relationship to the growth of two cancer cell lines.

We further carried out the *in vivo* efficacy studies of PEC. The results revealed that PEC exhibited strong anti-tumor activities against H22 hepatoma and S-180 sarcoma cells in mice, showing a significant dose-effect relationship. The inhibition rates against H22 and S-180 tumor growth in the highest dose group were 37.25 and 64.38%, respectively. PEC also exerted significantly inhibitory effects on transplanted SGC-7901 tumor in nude mice, and the tumor inhibition effect was proportional to the dose of PEC. The relative tumor inhibition rate against SGC-7901 tumor was 52.4% in the highest dose group.

In addition, we also performed preliminary studies to examine the effects of PEC on cell cycle progression, angiogenesis, and immune function. One of the key reasons that tumors can proliferate in an unrestrained manner is because of the malfunctioning of the cell cycle regulation mechanism, leading to uncontrolled cell growth. Tumors are sometimes considered to be fallout of a cell cycle disorder, resulting from uncontrolled cell growth, caused by genetic changes. Results of this study indicated that the proportion of cells in G1 phase gradually decreased, while the proportion of cells in G2 phase gradually increased by PEC. SGC-7901 cells were blocked in the G2 phase and a significant dose-effect relationship was observed.

Since Folkman et al. proposed the theory of tumor angiogenesis in 1971 (5), tumor growth and metastasis have been thought to be closely related to the formation of neovascularization. The restriction of blood vessel formation plays an important role in inhibiting the invasion and metastasis of solid tumors. VEGF, a vascular permeability factor (6), is a major factor in the process of human tumor angiogenesis. VEGF is a specific endothelial cell mitogen, which can promote the development of neovascularization *in vivo*. When cancer occurs, the expression of VEGF is significantly upregulated. Nayak et al. analyzed the expression of VEGF and MVD and concluded that MVD was related to the expression of VEGF (7). Therefore, drugs acting on VEGF have broad prospects for development. In this study, high dose PEC significantly inhibited the expression of VEGF in tumor cells, suggesting that PEC may play an important role in blocking angiogenesis by down-regulating the expression of VEGF and thereby affecting proliferation, growth, migration, and vascular permeability of vascular endothelial cells. However, whether the ability of PEC to inhibit tumor growth and angiogenesis is related to the improvement of other tumor microenvironmental factors is not fully understood and needs further study.

Lastly, the body's immune response includes cellular immunity and humoral immunity. B-cell mediated humoral immunity is an antigen-antibody reaction; the antigen-antibody complex can neutralize endotoxin and prevent infection caused by microorganisms and viruses. Cellular immunity is mainly carried out by T and NK cells. T cells can directly kill tumor cells, and produce a large number of lymphokines, including macrophage migration factors, lymphotoxin, transfer factors, and interferons. These lymphokines not only promote the proliferation and differentiation of immune cells, but also enhance phagocytosis and killing function of macrophages. Therefore, they play an important role in the anti-tumor process (8, 9). In this study, the immune activity of T and B cells was detected by the proliferative response of lymphocytes. Con A could stimulate T cell proliferation, and LPS could stimulate B cell proliferation. Results showed that the proliferative abilities of T and B lymphocytes of mice were enhanced after PEC administration. T and B lymphocytes in the highest dose group increased significantly (*P* < 0.05), showing a dose-effect relationship.

Inhibition of tumors requires the interaction between the specific and non-specific immune response. Non-specific immune response refers to the mechanisms of non-specific defense against antigen invasion, including phagocytosis, inflammation, barrier function etc., which also play an important role in the specific immune response process. NK cells and CD8^+^ CTL represent two major types of cytotoxic lymphocytes (10, 11), which can kill homogeneous and homologous pathogenic cells among infected cells, such as cancer cells. Therefore, they play an important role in the anti-tumor process (12). NK cells can inhibit the proliferation and differentiation of activated B cells, regulate the immune response by secreting cytokines such as IL-2 and interferon, and enhance immune surveillance. Therefore, NK cells are considered to be the first barrier of the body's immune defense system (13–15). The activity of NK cells is determined by routine cell immune response assays *in vitro*, and the anti-tumor activity of drugs can also be effectively determined. This study revealed that high dose PEC could significantly enhance the activity of NK cells and CTL in S-180 tumor-bearing mice and showed a dose-effect relationship. The activity of NK cells and CTL in the CTX control group was significantly lower than that in the tumor-bearing control group.

IL-2 is a polypeptide cytokine, produced by activated T cells which augment the immune functions of various immunocompetent cells. It exerts anti-tumor effects by activating tumor killing cells such as NK cells, CTL and lymphocyte activated killing cells (LAK). Specifically, it regulates the activation and proliferation of NK cells, promotes proliferation and differentiation of activated B cells, and enhances the killing effect of monocyte macrophages on tumor cells. At the same time, IFN-γ and other lymphocytes, secreted by NK cells activated by IL-2 can further enhance the anti-tumor activities of monocyte macrophages and NK cells (16–18). In this study, we found that IL-2 production by spleen cells of S-180 tumor-bearing mice was enhanced with increased PEC dose. Further, IL-2 secretion by spleen cells of mice in the high dose group was significantly higher than that in the model group.

Th1 type immune response is mediated by Th1 helper cells that mainly produce cytokines IL-2, TNF-β, IFN-γ, and antibody subclasses, IgG2a and IgG2b. It also participates in the cellular immunity by mediating immune responses related to cytotoxicity and local inflammation. The Th2 type immune response participates in the humoral immunity, mainly by producing cytokines (IL-4, IL-5, and IL-10), antibodies (IgG1 and IgA), and by stimulating B cell proliferation for antibody production. We have found that CTX could significantly reduce serum levels of tumor antigen specific IgG, IgG2a, and IgG2b antibodies, while PEC could increase the serum levels of these antibodies in tumor-bearing mice, especially in the high dose group. These results suggest that PEC primarily stimulates the Th1 type immune response *in vivo*.

In summary, we aimed at demonstrating the broad spectrum anticancer activities of PEC in this study. We have chosen seven cell lines *in vitro* studies. The results showed that PEC could inhibit the growth of all tumor cells. Among them, PC-3, PANC-1, and SGC-7901 had strong growth inhibition, the inhibition rate was more than 80%. Then, we chose the most common clinical cancer of gastric cancer (SGC-7901) as the research object. Taking this gastric cancer cell line as the representative, we discussed the anti-cancer mechanism of PEC by investigating its effects on apoptosis, cell cycle progression, and angiogenesis. In addition, we studied the effects of PEC on the immune function of S-180 sarcoma mice. In future studies, we will focus on the molecular mechanisms of PEC, which are critical for developing PEC as an anticancer drug.

## Conclusions

Our research team evaluated the *in vitro* and *in vivo* anticancer activity of ethyl acetate extracts from *P. perfoliatum* L. and further obtained the most active component PEC by activity-guided isolation. In the present study, we demonstrated the *in vitro* and *in vivo* antitumor effects of PEC as well as its mechanisms of action. Our results have shown that PEC inhibits cancer cell proliferation, induces apoptosis, and arrest cells at G2 phase. Furthermore, it has been found to reduce the expression of VEGF and MVD in tumor tissues and stimulate the proliferation of T and B lymphocytes in tumor-bearing mice. Moreover, it has also been shown to enhance the activity of NK cells and CTL, promote IL-2 secretion by spleen cells of mice, and increase the serum levels of IgG, IgG2a, and IgG2b antibodies in tumor-bearing mice. Taken together, PEC has shown promising anticancer activity *in vitro* and *in vivo* and could be developed as a therapeutic agent in future studies.

## Data Availability

All data generated or analyzed during this study are included in this published article (and its Supplementary Material Files).

## Author Contributions

QL and PH conceived and designed the experiments. FT, XG, and XF performed the experiments. HJ analyzed the data. QL wrote the paper. HJ revised the manuscript. All authors have reviewed the final manuscript.

### Conflict of Interest Statement

The authors declare that the research was conducted in the absence of any commercial or financial relationships that could be construed as a potential conflict of interest.
