# Supplementary material for: Antitumor Effects and Related Mechanisms of Ethyl Acetate Extracts of Polygonum perfoliatum L
Source: Front Oncol. 2019 Jul 4;9:578. doi: 10.3389/fonc.2019.00578 (PMC6621420; doi:10.3389/fonc.2019.00578)
Supplement: Supplementary file 1 [file Table_1.doc]

**Antitumor Effects and Related Mechanisms of Ethyl Acetate Extracts of *Polygonum perfoliatum* L.**

**Qinglin Li^1,2^*, Xiaoxuan Fu^2^, Xinyang Ge^3^, Feng Tao^4^ *, Ping Huang^2^*, Minghua Ge^2^*, Hongchuan Jin ^1^ ***

^1^Sir Run Run Shaw hospital, Medical School of Zhejiang University, Hangzhou, China

^2^Zhejiang Cancer Hospital, Hangzhou, China

^3^Heartland Christian School Columbiana, OH, USA

^4^Hangzhou Medical College, Hangzhou, China

*** Correspondence:**Feng Tao

[12320934@qq.com](mailto:12320934@qq.com)

Qinglin Li

qinglin200886@126.com

Ping Huang

[huangping1841@zjcc.org.cn](mailto:huangping1841@zjcc.org.cn)

Minghua Ge

[gemingh@163.com](mailto:gemingh@163.com)

Hongchuan Jin

[jinhc@zju.edu.cn](mailto:jinhc@zju.edu.cn)

Keywords: Antitumor, Effects, Mechanisms, *Polygonum perfoliatum* L.,PEC.

| S-Table 1 Inhibitory effect of PEC on H22 xenograft tumor in mice (±S, n=10) | | | | | |
| --- | --- | --- | --- | --- | --- |
| Group | Administration | Mice weight (g) | | Tumor weight (g) | Inhibition rate (%) |
|  |  | Beginning | Ending |  |  |
| Model | ig. | 21.6±1.0 | 28.6±2.4 | 1.07±0.19 | — |
| CTX25 mg/kg | ip. | 21.3±0.9 | 25.2±2.1 | 0.51±0.14 | 52.34** |
| PEC3.5 mg/kg | ig. | 21.5±1.1 | 28.1±2.4 | 0.87±0.07 | 18.69 |
| PEC 7 mg/kg | ig. | 21.2±0.8 | 27.6±2.3 | 0.69±0.28 | 35.51* |
| PEC 14 mg/kg | ig. | 21.7±0.9 | 27.7±1.9 | 0.61±0.24 | 37.25* |

Compared with model group：**P< 0.05*，***P< 0.01*

| S- Table 2 Inhibitory effect of PEC on H22 xenograft tumor in mice (±S, n=10） | | | | | |
| --- | --- | --- | --- | --- | --- |
| Group | Administration | Mice weight (g) | | Tumor weight (g) | Inhibition rate (%) |
|  |  | Beginning | Ending |  |  |
| Model | ig. | 22.3±1.1 | 28.1±2.3 | 1.60±0.21 | — |
| CTX25 mg/kg | ip. | 22.1±1.0 | 25.0±2.1 | 0.81±0.16 | 49.38** |
| PEC 3.5 mg/kg | ig. | 22.1±1.0 | 27.4±1.7 | 0.98±0.25 | 38.75* |
| PEC 7 mg/kg | ig. | 21.9±0.9 | 26.8±2.1 | 0.77±0.19 | 51.88** |
| PEC 14 mg/kg | ig. | 22.0±1.1 | 26.6±2.4 | 0.57±0.24 | 64.38** |

Compared with model group：**P＜0.05*，***P＜0.01*

| S- Table 3 Inhibitory effect of PEC on transplanted human tumor SGC-7901 in nude mice (±S, n=10) | | | | | | |
| --- | --- | --- | --- | --- | --- | --- |
| Group | Administration | Mice weight (g) | | Beginning/Ending Tumor volume (mm^3^) | RTV | T/C (%) |
|  |  | Beginning | Ending |  |  |  |
| Model | ig | 21.4±1.1 | 28.6±2.4 | 243.2/4036.7 | 16.6 | 100 |
| CTX 25 mg/kg | ip | 21.2±0.9 | 25.2±2.1 | 197.6/1142.7 | 5.8 | 34.9** |
| PEC 3.5 mg/kg | ig | 21.6±1.1 | 28.1±2.4 | 257.4/3592.6 | 13.9 | 83.7 |
| PEC 7 mg/kg | ig | 21.8±0.9 | 27.6±2.3 | 173.6/1984.3 | 11.4 | 68.7* |
| PEC 14 mg/kg | ig | 21.3±1.1 | 27.7±1.9 | 218.3/1891.6 | 8.7 | 52.4* |

Compared with model group：**P＜0.05,* ***P＜0.01*
